# Supplementary material for: A natural mutation between SARS-CoV-2 and SARS-CoV determines neutralization by a cross-reactive antibody
Source: bioRxiv. 2020 Sep 21:2020.09.21.305441. Preprint. [Version 1] doi: 10.1101/2020.09.21.305441 (PMC7523114; doi:10.1101/2020.09.21.305441)
Supplement: 1 [file NIHPP2020.09.21.305441-supplement-1.pdf]

bioRxiv preprint doi: https://doi.org/10.1101/2020.09.21.305441. this version posted September 21, 2020. The copyright holder for this preprint (which was not certified by peer review) is the author/funder. It is made available under a CC-BY 4.0 International license.

Supplementary Table 1. X-ray data collection and refinement statistics.

**Data collection**	
Beamline	SSRL 12-2
Wavelength (Å)	0.97946
Space group	C 1 2 1
Unit cell parameters (Å and °)	a=265.7, b=59.9, c=51.7, β=99.8
Resolution (Å)	50.0–2.70 (2.76–2.70)[a]
Unique reflections	21,547 (2,021)[a]
Redundancy	6.7 (5.5)[a]
Completeness (%)	100.0 (100.0)[a]
$<I/\sigma_I>$	14.7 (1.0)[a]
R_{sym}[b] (%)	9.2 (86.1)[a]
R_{pim}[b] (%)	5.4 (54.8)[a]
$CC_{1/2}$[c] (%)	99.4 (74.4)[a]
**Refinement statistics**	
Resolution (Å)	45.0–2.70
Reflections (work)	21,501
Reflections (test)	1,011
R_{cryst}[d] / R_{free}[e] (%)	22.2 / 27.6
No. of atoms	4,872
Macromolecules	4,795
Glycans	42
Solvent	30
Average *B*-value (Å^2)	80
Macromolecules	80
RBD	104
Fab	70
Glycans	30
Solvent	60
Wilson *B*-value (Å^2)	64
**RMSD from ideal geometry**	
Bond length (Å)	0.005
Bond angle (°)	1.17
**Ramachandran statistics (%)**	
Favored	95.6
Outliers	0.16
**PDB code**	7JN5

[a] Numbers in parentheses refer to the highest resolution shell.

[b] $R_{sym} = \Sigma_{hkl} \Sigma_i | I_{hkl,i} - <I_{hkl}> | / \Sigma_{hkl} \Sigma_i I_{hkl,i}$ and $R_{pim} = \Sigma_{hkl} (1/(n-1))^{1/2} \Sigma_i | I_{hkl,i} - <I_{hkl}> | / \Sigma_{hkl} \Sigma_i I_{hkl,i}$, where $I_{hkl,i}$ is the scaled intensity of the ith measurement of reflection h, k, l, $<I_{hkl}>$ is the average intensity for that reflection, and *n* is the redundancy.

[c] $CC_{1/2}$ = Pearson correlation coefficient between two random half datasets.

[d] $R_{cryst} = \Sigma_{hkl} | F_o - F_c | / \Sigma_{hkl} | F_o | \times 100$, where F_o and F_c are the observed and calculated structure factors, respectively.

[e] R_{free} was calculated as for R_{cryst}, but on a test set comprising 5% of the data excluded from refinement.

bioRxiv preprint doi: https://doi.org/10.1101/2020.09.21.305441. this version posted September 21, 2020. The copyright holder for this preprint (which was not certified by peer review) is the author/funder. It is made available under a CC-BY 4.0 International license.

724 **Supplementary Table 2. Cryo-EM data collection and refinement statistics.**

**Map**	**SARS-CR3022Fab Class 1**	**SARS-CR3022Fab Class 2**	**SARS-CR3022Fab Class 3**	**SARS-CR3022Fab Class 4**
EMDB	Pending	Pending	Pending	Pending
**Data collection**				
Microscope	FEI Talos Arctica	FEI Talos Arctica	FEI Talos Arctica	FEI Talos Arctica
Voltage (kV)	200	200	200	200
Detector	Gatan K2 Summit	Gatan K2 Summit	Gatan K2 Summit	Gatan K2 Summit
Recording mode	Counting	Counting	Counting	Counting
Nominal magnification	36,000	36,000	36,000	36,000
Movie micrograph pixelsize (Å)	1.15	1.15	1.15	1.15
Dose rate (e^-/[(camera pixel)*s])	5.6	5.6	5.6	5.6
Number of frames per movie micrograph	47	47	47	47
Frame exposure time (ms)	250	250	250	250
Movie micrograph exposure time (s)	11.7	11.7	11.7	11.7
Total dose (e^-/Å^2)	50	50	50	50
Defocus range (µm)	-0.4 to -1.6	-0.4 to -1.6	-0.4 to -1.6	-0.4 to -1.6
**EM data processing**				
Number of movie micrographs	2952	2952	2952	2952
Number of molecular projection images in map	17,472	28,821	34,803	31,645
Symmetry	C1	C1	C1	C1
Map resolution (FSC 0.143; Å)	6.83	6.24	6.42	6.15
Map sharpening B-factor (Å^2)	-164.6	-147.4	-120.5	-138.1

725

bioRxiv preprint doi: https://doi.org/10.1101/2020.09.21.305441. this version posted September 21, 2020. The copyright holder for this preprint (which was not certified by peer review) is the author/funder. It is made available under a CC-BY 4.0 International license.

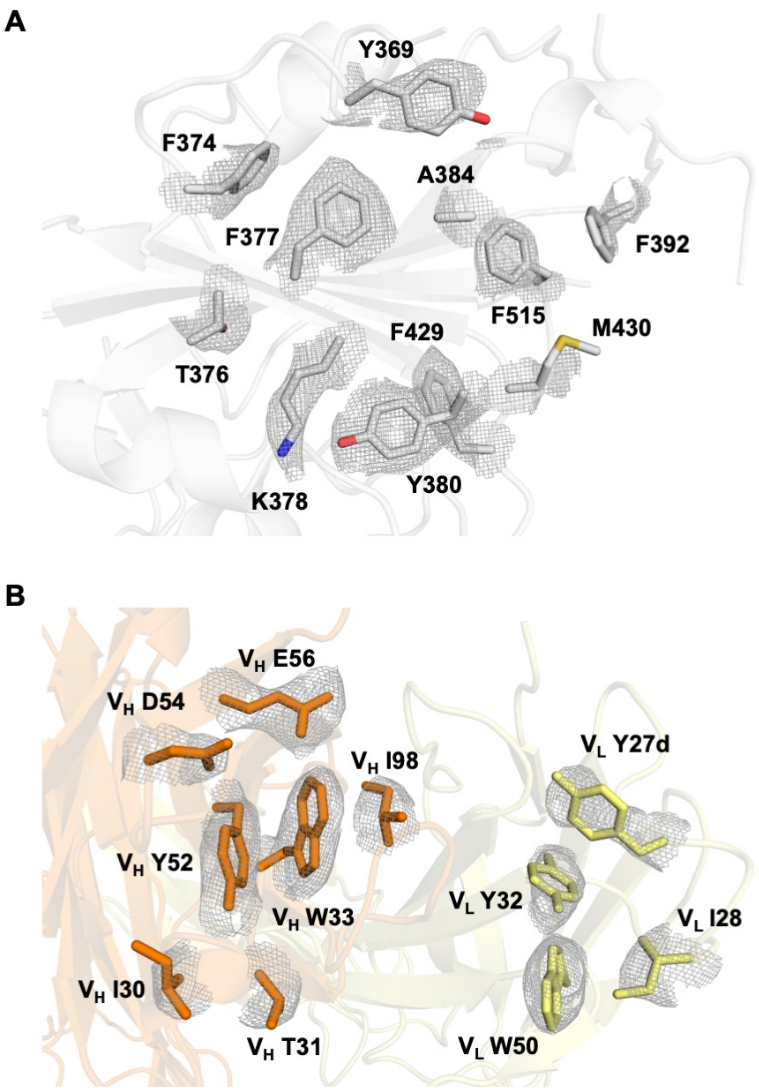

Supplementary Figure 1. X-ray electron density maps for epitope and paratope regions of SARS CoV RBD with Fab CR3022. (A) Final 2Fo-Fc electron density maps for the side chains in the epitope region of SARS-CoV-2 contoured at 1 σ. **(B)** Final 2Fo-Fc electron density maps for the paratope region of CR3022 contoured at 1 σ. The heavy chain is colored in orange, and light chain in yellow. Epitope and paratope residues are labeled.

bioRxiv preprint doi: https://doi.org/10.1101/2020.09.21.305441. this version posted September 21, 2020. The copyright holder for this preprint (which was not certified by peer review) is the author/funder. It is made available under a CC-BY 4.0 International license.

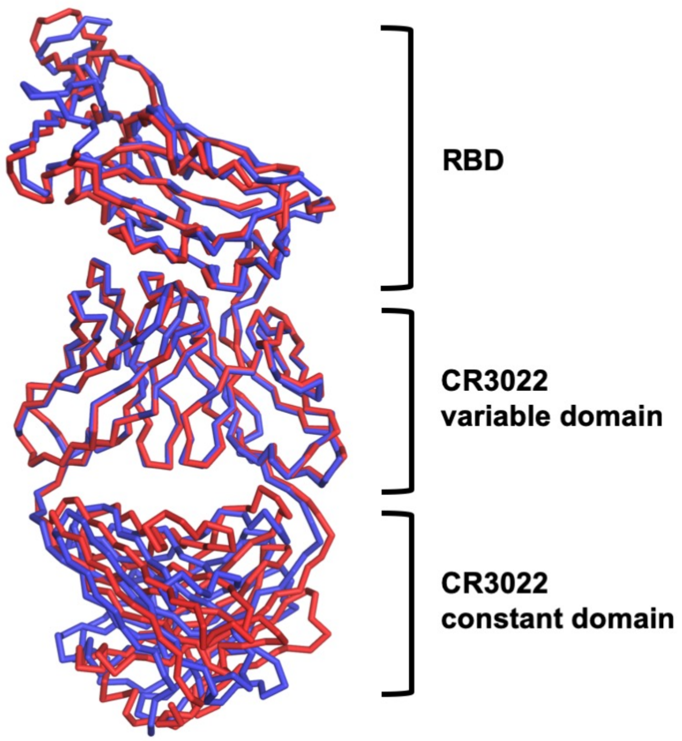

Supplementary Figure 2. Structural alignment of CR3022-bound SARS-CoV RBD and CR3022-bound SARS-CoV-2 RBD. Structure of CR3022 in complex with SARS-CoV RBD (this study) is aligned to that with SARS-CoV-2 RBD (PDB 6W41). Structural alignment was performed using CR3022 heavy chain variable domain. Red: CR3022 in complex with SARS-CoV RBD. Blue: CR3022 in complex with SARS-CoV-2 RBD.

bioRxiv preprint doi: https://doi.org/10.1101/2020.09.21.305441. this version posted September 21, 2020. The copyright holder for this preprint (which was not certified by peer review) is the author/funder. It is made available under a CC-BY 4.0 International license.

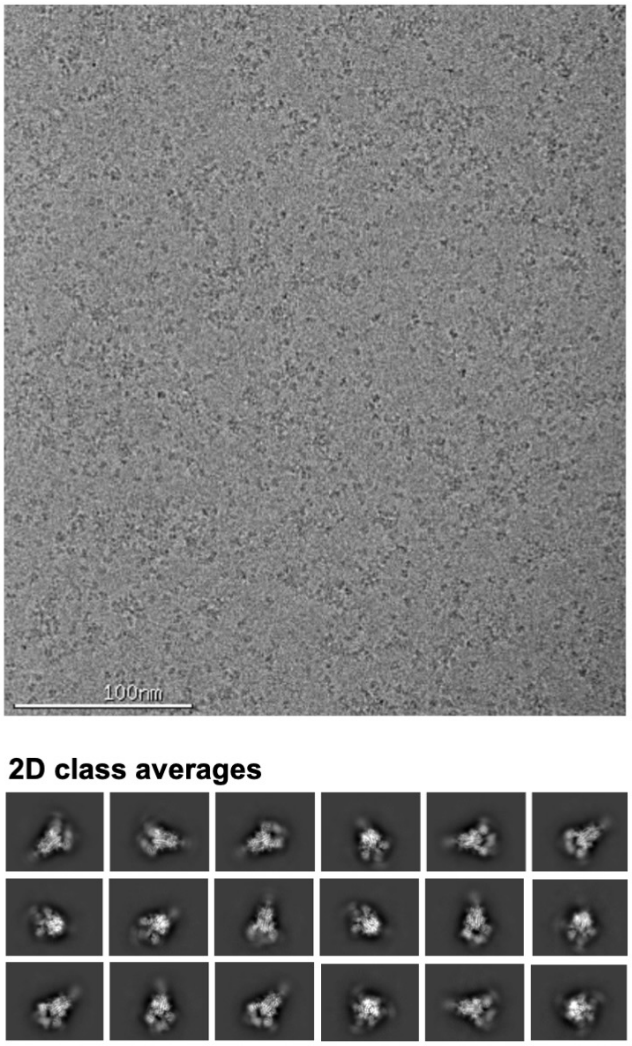

Supplementary Figure 3. Representative cryo-electron micrograph and 2D class averages of the SARS-CoV spike in complex with CR3022 Fab. The top panel shows a representative cryo-electron micrograph of the SARS-CoV spike complexed with CR3022 Fab, whereas the bottom panels show the 2D class averages.

bioRxiv preprint doi: https://doi.org/10.1101/2020.09.21.305441. this version posted September 21, 2020. The copyright holder for this preprint (which was not certified by peer review) is the author/funder. It is made available under a CC-BY 4.0 International license.

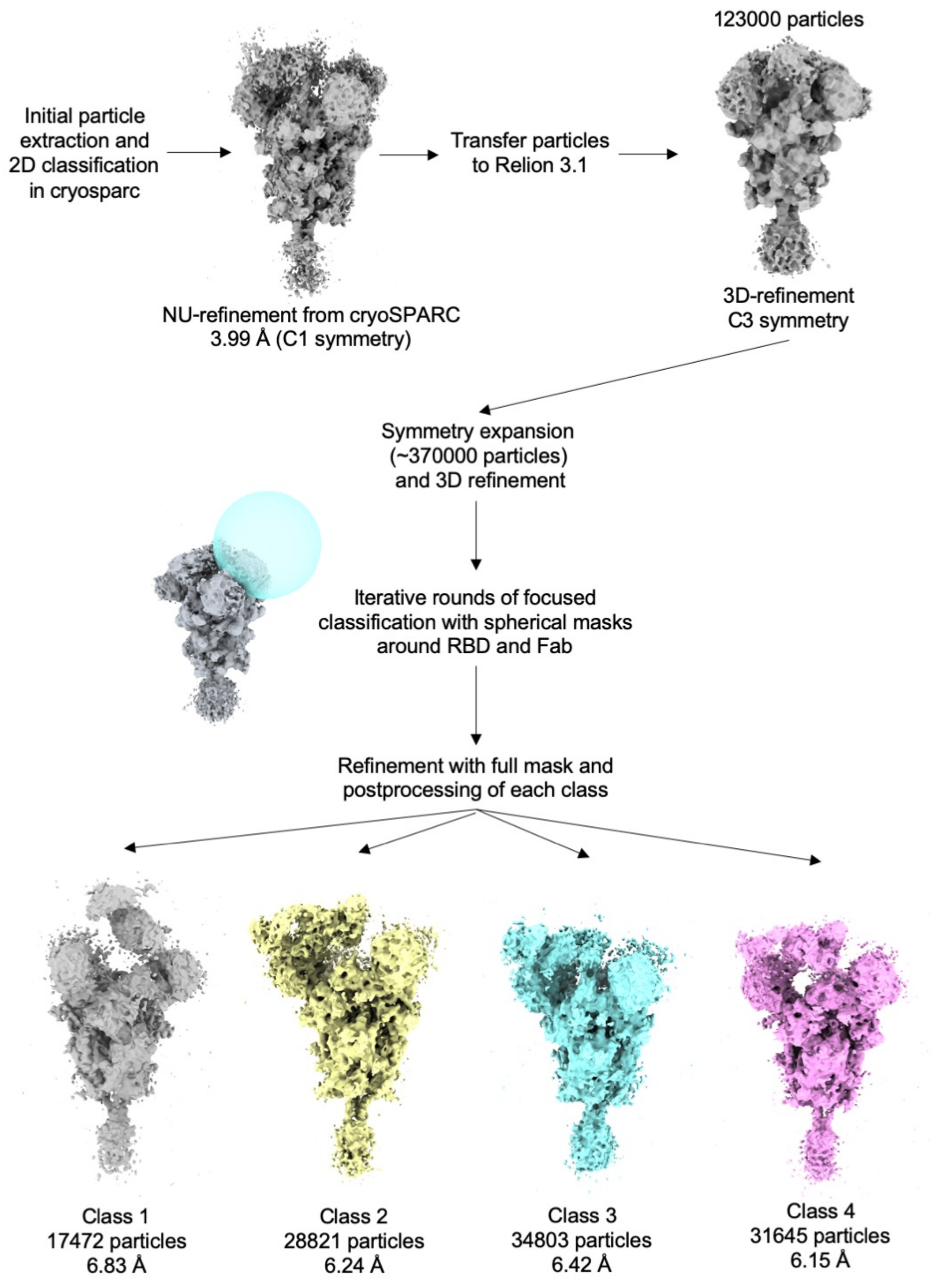

Supplementary Figure 4. Workflow for cryo-EM data processing. Four 3D class averages of complex of the SARS-CoV spike and CR3022 were found during data processing.

bioRxiv preprint doi: https://doi.org/10.1101/2020.09.21.305441. this version posted September 21, 2020. The copyright holder for this preprint (which was not certified by peer review) is the author/funder. It is made available under a CC-BY 4.0 International license.

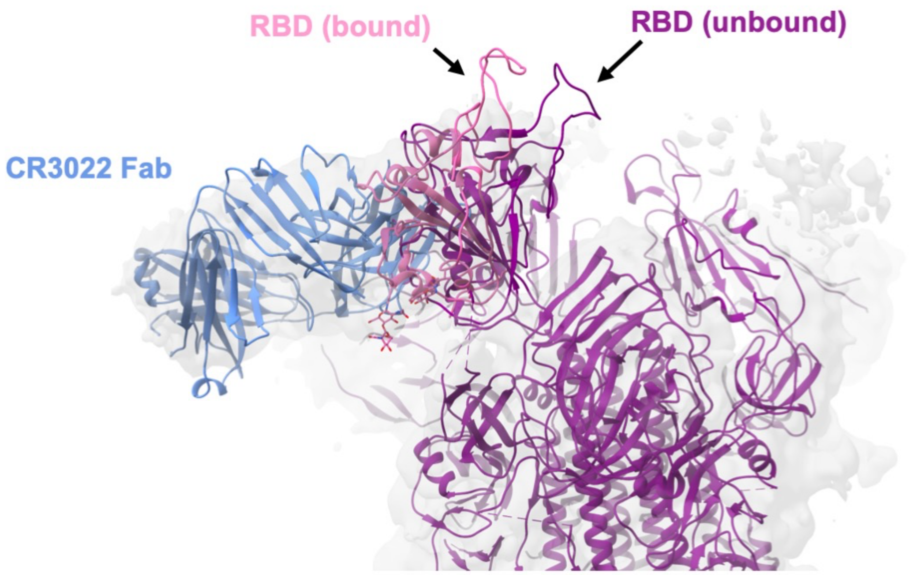

Supplementary Figure 5. Comparison of conformations of CR3022-bound and unbound RBDs. The conformation of CR3022-bound RBD in class 2 and 4 is compared to the conformation of RBD on an unliganded SARS-CoV S protein (PDB 6ACD) [35].

bioRxiv preprint doi: https://doi.org/10.1101/2020.09.21.305441. this version posted September 21, 2020. The copyright holder for this preprint (which was not certified by peer review) is the author/funder. It is made available under a CC-BY 4.0 International license.

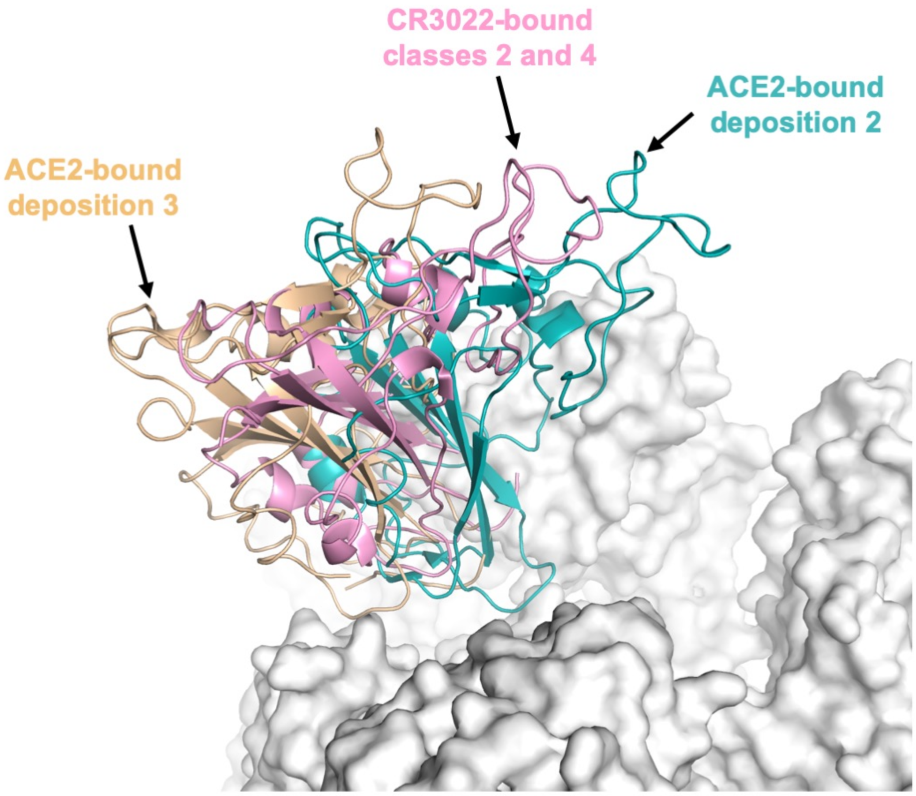

Supplementary Figure 6. Comparison of conformations of CR3022-bound and ACE2-bound RBDs. The conformation of CR3022-bound RBD in class 2 and 4 is compared to that of depositions 2 and 3 of ACE2-bound RBD (PDB 6ACJ and 6ACK, respectively) [35].
